# Supplementary material for: A Critical View of the Application of the APEX Software (Aqueous Photochemistry of Environmentally-Occurring Xenobiotics) to Predict Photoreaction Kinetics in Surface Freshwaters
Source: Molecules. 2019 Dec 18;25(1):9. doi: 10.3390/molecules25010009 (PMC7017383; doi:10.3390/molecules25010009)
Supplement: Supplementary file 1 [file molecules-25-00009-s001.zip › 3.2.4_gcc-4.4.0.zip!/3.2.4_gcc-4.4.0/3.2.4_gcc-4.4.0/doc/octave/html/liboctave/index.html]

Octave C++ Classes


Copyright (C) 1996, 1997 John W. Eaton.

Permission is granted to make and distribute verbatim copies of
this manual provided the copyright notice and this permission notice
are preserved on all copies.

Permission is granted to copy and distribute modified versions of
this manual under the conditions for verbatim copying, provided that
the entire resulting derived work is distributed under the terms of
a permission notice identical to this one.

Permission is granted to copy and distribute translations of this
manual into another language, under the above conditions for
modified versions.

## Table of Contents

- Octave C++ Classes- 1 Acknowledgements
    - Contributors to Octave
    - GNU GENERAL PUBLIC LICENSE- 2 A Brief Introduction to Octave- 3 Arrays
          - 3.1 Constructors and Assignment
          - 4 Matrix and Vector Operations- 5 Matrix Factorizations- 6 Ranges- 7 Nonlinear Functions- 8 Nonlinear Equations- 9 Optimization
                      - 9.1 Objective Functions- 9.2 Bounds- 9.3 Linear Constraints- 9.4 Nonlinear Constraints- 9.5 Quadratic Programming- 9.6 Nonlinear Programming
                      - 10 Quadrature
                        - 10.1 Collocation Weights
                        - 11 Ordinary Differential Equations- 12 Differential Algebraic Equations- 13 Error Handling- 14 Installation- 15 Bugs- Concept Index- Function Index

Next: Acknowledgements,
Previous: (dir),
Up: (dir)


---

## Octave C++ Classes

This manual documents how to use, install and port Octave's C++ class
library, and how to report bugs. It corresponds to Octave version
3.2.4.

- Acknowledgements- Copying- Introduction- Arrays- Matrix and Vector Operations- Matrix Factorizations- Ranges- Nonlinear Functions- Nonlinear Equations- Optimization- Quadrature- Ordinary Differential Equations- Differential Algebraic Equations- Error Handling- Installation- Bugs- Concept Index- Function Index

--- The Detailed Node Listing ---

Acknowledgements

- Contributors: People who contributed to developing of Octave.

Arrays

- Constructors and Assignment

Optimization

- Objective Functions- Bounds- Linear Constraints- Nonlinear Constraints- Quadratic Programming- Nonlinear Programming

Quadrature

- Collocation Weights
